# Prospective diagnostic accuracy study of history taking and physical examination for adults with vertigo in general practice: study protocol

**DOI:** 10.1136/bmjopen-2024-085715

**Published:** 2024-04-02

**Authors:** Andrew Ross, Anna-Marie Rebecca Leemeyer, Tjasse D Bruintjes, Jochen W L Cals, Adolfo Bronstein, Roeland B van Leeuwen, Birgit Lissenberg-Witte, Vincent Alexander van Vugt, Sandra Rutgers, Otto R Maarsingh

**Affiliations:** 1 Department of General Practice, Amsterdam UMC Location VUmc, Amsterdam, The Netherlands; 2 Amsterdam Public Health Research Institute, Amsterdam, The Netherlands; 3 Department of Otorhinolaryngology—Head and Neck Surgery, Leiden University Medical Center, Leiden, The Netherlands; 4 Department of Neurology, Gelre Hospital, Apeldoorn, The Netherlands; 5 Department of Family Medicine, Maastricht University Care and Public Health Research Institute, Maastricht, The Netherlands; 6 Neuro-otology Unit, Division of Brain Sciences, Imperial College London, London, UK; 7 Department of Epidemiology and Data Science, Amsterdam UMC Locatie VUmc, Amsterdam, The Netherlands; 8 Patient association Hoormij NVVS, Houten, The Netherlands

**Keywords:** Primary Health Care, Primary Care, Adult otolaryngology

## Abstract

**Introduction:**

Vertigo is a prevalent and burdensome symptom. More than 80% of patients with vertigo are primarily treated by their general practitioner (GP) and are never referred to a medical specialist. Despite this therapeutic responsibility, the GP’s diagnostic toolkit has serious limitations. All recommended tests lack empirical evidence, because a diagnostic accuracy study on vestibular disorders (‘How well does test x discriminate between patients with or without target condition y?’) has never been performed in general practice. The VERtigo DIagnosis study aims to fill this gap.

**Methods and analysis:**

We will perform a diagnostic accuracy study on vertigo of primary vestibular origin in general practice to assess the discriminative ability of history taking and physical examination. We will compare all index tests with a respective reference standard. We will focus on five target conditions that account for more than 95% of vertigo diagnoses in general practice: (1) benign paroxysmal positional vertigo, (2) vestibular neuritis, (3) Ménière’s disease, (4) vestibular migraine (VM) and (5) central causes other than VM. As these five target conditions have a different pathophysiology and lack one generally accepted gold standard, we will use consensus diagnosis as a construct reference standard. Data for each patient, including history, physical examination and additional tests as recommended by experts in an international Delphi procedure, will be recorded on a standardised form and independently reviewed by a neurologist and otorhinolaryngologist. For each patient, the reviewers have to decide about the presence/absence of each target condition. We will calculate sensitivity, specificity, predictive values, likelihood ratios and diagnostic ORs, followed by decision rules for each target condition.

**Ethics and dissemination:**

The study obtained approval from the Vrije Universiteit Medical Center Medical Ethical Review Committee (reference: 2022.0817—NL83111.029.22). We will publish our findings in peer-reviewed international journals.

**Trial registration number:**

ISRCTN97250704.

**STRENGTHS AND LIMITATIONS OF THIS STUDY**

- The VERtigo DIagnosis study is a prospective diagnostic accuracy study that addresses the scientific gap of absent empirical evidence on the diagnostic value of history taking and physical examination for patients with vertigo in general practice.
- In order to improve the diagnostic toolkit of general practitioners, we will use data for individual diagnostic tests to construct easy-to-use diagnostic algorithms for the most prevalent causes of vertigo.
- Although this diagnostic accuracy study focuses on five target conditions—accounting for more than 95% of vertigo diagnoses in general practice, the study does not cover all possible causes of vertigo.

## Introduction

According to the International Classification of Vestibular Disorders (ICVD) of the Bárány Society, vertigo is “the sensation of self-motion when no self-motion is occurring or the sensation of distorted self-motion during an otherwise normal head movement”.^1 2^ It is a common symptom that increases with age, with a prevalence ranging from 5% in patients aged 18 or older to >30% in patients aged 75 or older.^3 4^ The burden of illness is substantial: four out of five patients with vertigo report severely impairing symptoms, leading to medical consultation, interruption of daily activities and/or avoidance of leaving the house.^4^ Moreover, medical consumption and decreased productivity form a large economic burden.^5^

Each year, a Dutch general practitioner (GP) with a standard practice list size (n=2095 patients) sees about 35 patients with vertigo and/or dizziness, of whom 17 patients with vertigo.^6^ More than 80% of patients experiencing vertigo in the Netherlands, the UK and the USA are primarily treated by their GP and are never referred to a medical specialist.^7–9^ The symptoms of these patients are predominantly caused by peripheral vestibular disease, like vestibular neuritis, benign paroxysmal positional vertigo (BPPV) or Ménière’s disease.^7 10^ Sometimes, vertigo in general practice is caused by vestibular migraine (VM)—a benign diagnosis most GPs are not familiar with.^11^ Incidentally, vertigo in general practice has a central and serious cause such as a cerebrovascular event.^7 10^ Despite the fact that GPs treat the large majority of patients with vertigo, their ‘diagnostic toolkit’ has serious limitations. First, there is no empirical evidence on the diagnostic value of history taking and physical examination for patients with vertigo in general practice, because diagnostic accuracy studies on conditions that may cause vertigo have never been performed in a primary care setting.^12 13^ Second, all recommendations for diagnostic testing in (inter)national guidelines are based on consensus and derivative suboptimal evidence from secondary and tertiary care.^6 14–18^ In particular, the quality of the empirical evidence for the recommended Dix-Hallpike manoeuvre is low due to selection bias (tertiary care patients not representative of patients seen in general practice), verification bias (only part of the study population received the reference test) and incorporation bias (reference test incorporates the result of the index test bias,^6 12 19–24^ while the quality of the empirical evidence for the recommended head impulse test is high but was performed among selected populations with patients not representative of general practice, leaving its value for GPs unclear.^6 12 19 25^

Furthermore, previous research suggests that GP diagnosis can be improved: GPs were able to provide a final diagnosis for 60%–64% of the older patients with vertigo/dizziness,^8 26^ whereas an expert panel could provide a diagnosis for 92% of the patients.^27^ Also, GPs hardly (1%–3%) diagnosed other contributory causes,^8 26^ whereas the panel identified other contributory causes in 46% of the patients.^27^

Finally, diagnostic errors sometimes have serious consequences. Whereas vertigo and dizziness are the symptoms most tightly linked to missed stroke, no less than 9% of cerebrovascular events are missed at initial emergency department presentation.^28^ Accurate and efficient diagnosis for these patients, although low-prevalent in primary care, may save lives and reduce costs through prompt and appropriate treatment.^29^

We hypothesise that the construction of a diagnostic algorithm for GPs may lead to faster and more targeted treatment, less diagnostic imaging and referral to the emergency department and/or hospital, less prescribing of antivertigo drugs and improvement of the overall outcome for patients with vertigo in general practice.

### Overarching VERtigo DIagnosis (VERDI) project

**Figure 1:**
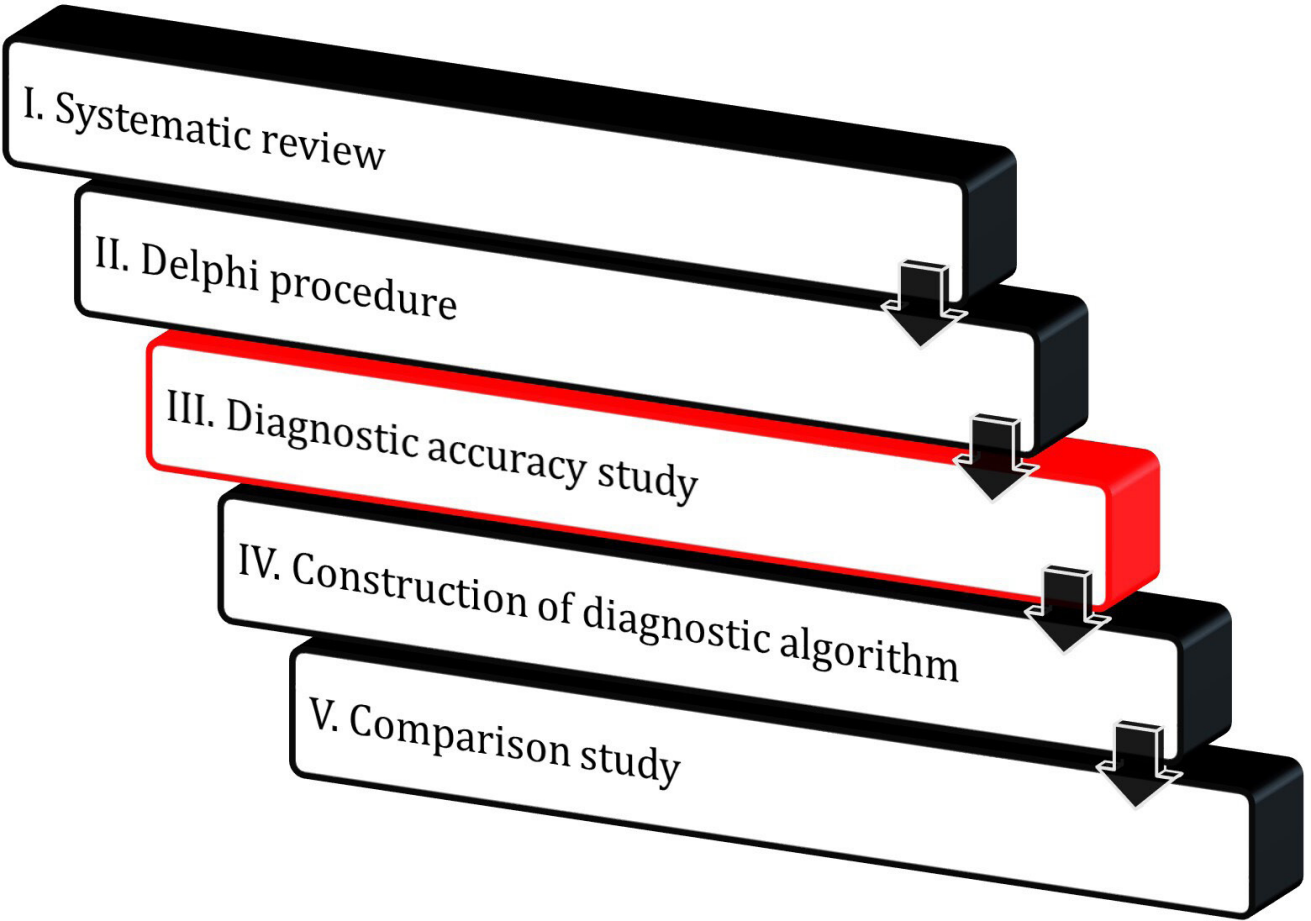
The five phases of the VERtigo DIagnosis project; the focus of the current study protocol, that is, the diagnostic accuracy study, is highlighted in red.

The VERDI project (also relating to Giuseppe Verdi, the Italian composer who experienced frequent episodes of vertigo and died of stroke) is a five-phase study. The current study is a study protocol for phase III of the VERDI study, that is, the diagnostic accuracy study (see figure 1). The design and objective of the other phases of VERDI are outlined below to explain the role of the current study within VERDI:

- To assess and summarise the existing evidence on the accuracy of tests for diagnosing causes of vertigo in general practice (Systematic review);
- To determine which diagnostic tests should be investigated in a diagnostic accuracy study for patients with vertigo in general practice (Delphi Procedure);
- To investigate the diagnostic accuracy of history taking and physical examination for patients with vertigo in general practice (Diagnostic accuracy study);
- To construct an easy-to-use diagnostic algorithm for diagnosing causes of vertigo in general practice (Construction of diagnostic algorithm);
- To compare the diagnostic accuracy of GP judgement with the constructed diagnostic algorithm (Comparison study).

## Methods and analysis

### Aims and objectives

The main objective is to investigate the diagnostic accuracy of history taking and physical examination for patients with vertigo of primary vestibular origin in general practice.

### Study design

We will perform a prospective diagnostic accuracy study in general practice (‘How well does test x discriminate between patients with or without target condition y?’). Index tests include both questions during history taking (eg, ‘Is your vertigo accompanied by a headache?’) and diagnostic tests during the physical examination (eg, Dix-Hallpike manoeuvre). Target conditions: (1) BPPV, (2) vestibular neuritis, (3) Ménière’s disease, (4) VM and (5) central causes of vertigo other than VM. We will use the Standards for Reporting of Diagnostic Accuracy 2015 checklist to provide accurate and complete reporting of the results of our diagnostic accuracy study.^30^ Patient recruitment runs from March 2023 until February 2026; the overall study end date is 31 December 2027.

### Participants

**Figure 2:**
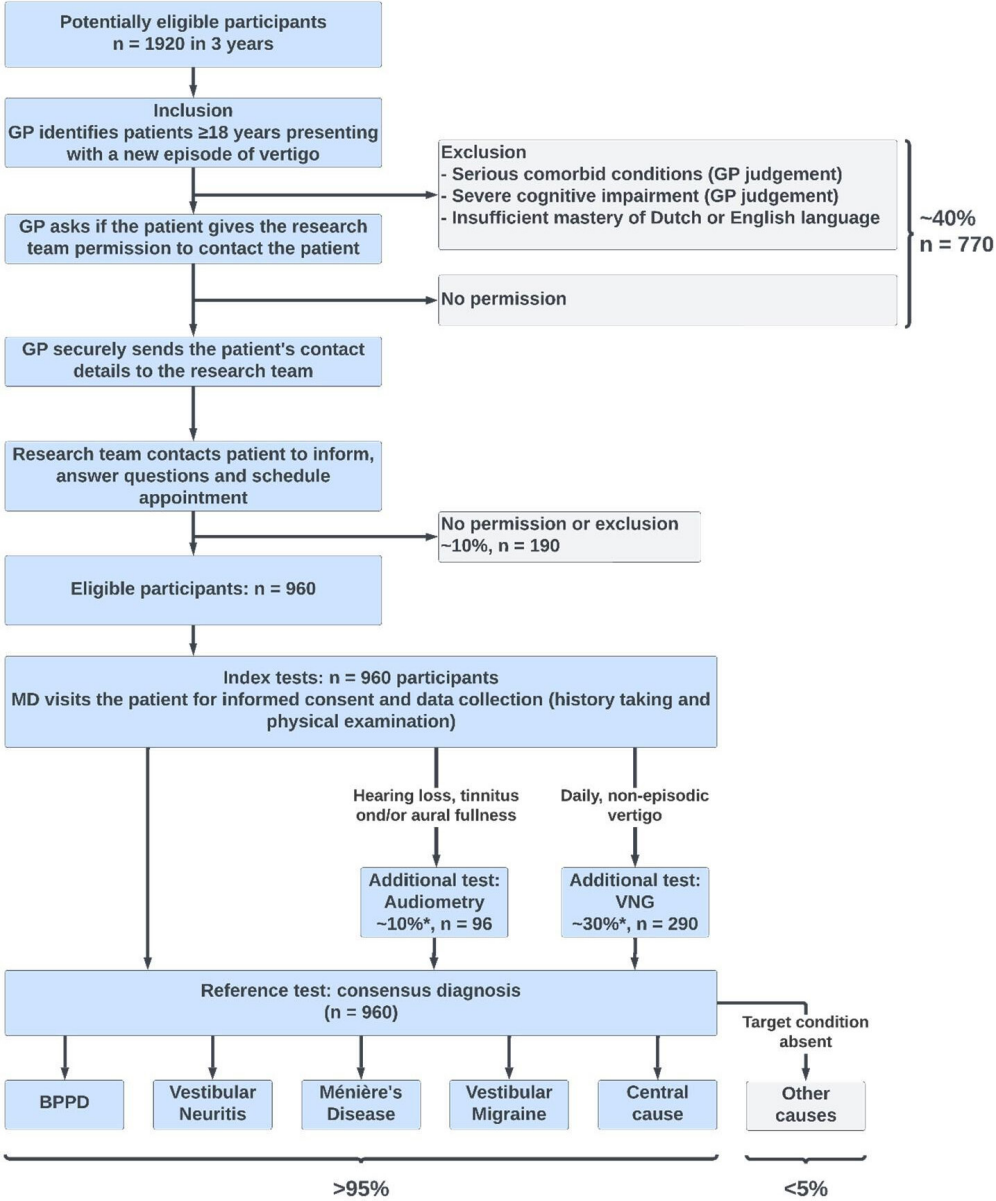
Flow chart of the VERtigo DIagnosis diagnostic accuracy study. GP, general practitioner; VNG, videonystagmography. *The estimated number of additional tests reported here (~10% for audiometry and ~30% for VNG) is based on additional analyses of the DIEP study. (Reported estimates reproduced from Maarsingh *et al*.^27^) DIEP, Dizziness In Elderly Patients study.

We aim to recruit 960 patients from general practices in the Netherlands during 36 months (see figure 2). Eligible participants are adults aged 18 years and older presenting with vertigo—according to the definition of the ICVD of the Bárány Society^1 2^—in general practice. GPs are instructed to provide usual care, before, during and after enrolment of patients. At the end of a consultation, the GP will identify potential participants. The research team will approach potential participants by telephone and provide background information about the study, answer questions, check the eligibility criteria (see table 1), and, when the patient has the intention to participate, schedule an appointment for a home visit after 48 hours.

**Table 1:** Inclusion and exclusion criteria

Inclusion criteria	Exclusion criteria
Aged 18 years and over	Serious comorbid conditions that preclude participation in the VERDI study (judgement of patient’s GP)
New episode of vertigo and/or episodic vestibular syndrome	Severe cognitive impairment (judgement of patient’s GP)
Presentation of symptoms during (telephone) consultation or home visit	Insufficient mastery of Dutch and English language

GP, general practitioner; VERDI, VERtigo DIagnosis.

### Home visit

After successful enrolment, participants will be investigated during a planned home visit by study physicians with clinical experience who received a 3-hour training aimed at optimally performing the index tests according to the current guidelines. During the home visit, the trained study physicians will record all clinical data using an electronic case report form (see the Data collection section). When the study physician has entered the results of the index tests, the electronic case report form will automatically provide an overview of indicated additional diagnostic tests for this individual patient according to the current guidelines.^1 11 25 29 31 32^ In case of symptoms that warrant immediate referral (ie, ‘red flags’ like diplopia, dysphagia, ataxia, facial weakness or numbness, see table 2),^6 19^ the study physician will instantly contact the principal investigator of the study. If necessary, the study physician will contact the participant’s GP to discuss further clinical management/immediate referral.

**Table 2:** ‘Red flags’: serious conditions associated with acute vertigo and/or dizziness, for which GP assessment in the (very) short term or direct referral is necessary (the Dizziness Guideline of the Dutch College of GPs^6^)

Characteristics	Urgent condition(s)
Sudden dizziness with neurological symptoms, such as a combination of vertigo with dysarthria, diplopia, dysphagia or ataxia*; a paresis in one or both body halves, sensibility disorder in one or both body halves or homonymous hemianopia	Cerebral infarction, cerebral haemorrhage, transient ischaemic attack
Acute dizziness paired with simultaneously arisen acute *hearing loss*	Cerebral infarction, cerebral haemorrhage, transient ischaemic attack
Acute dizziness paired with acute, very severe headache or neck pain, whether or not after traumatic head injury	Subarachnoid haemorrhage, vertebral artery dissection
Lightheadedness paired with (almost) *falling/collapse* where a cardiac cause is suspected	(Serious) cardiac arrhythmias
Acute dizziness, headache, nausea, lethargy and prolonged stay in a closed room without ventilation	Carbon monoxide intoxication
Briefly existing dizziness in diabetes mellitus and abnormal behaviour or other indications of hypoglycaemia	Hypoglycaemia

*Extremity ataxia (abnormal finger-to-nose test, heel-to-shin test and diadochokinesis) is consistent with a central cause of dizziness. An abnormal (broad-based) gait pattern can occur with both central and vestibular causes and is therefore not distinctive.

GP, general practitioner.

#### Index tests

The index tests under investigation (see table 3) will include all diagnostic tests that are recommended by the Dizziness Guideline of the Dutch College of GPs.^6^ This list of tests will be complemented with diagnostic tests for vertigo that were assessed during a systematic review (phase I VERDI) and were deemed appropriate for use in general practice by experts in an international Delphi procedure (phase II). During history taking, we will record sociodemographic characteristics (age, sex, education, partner status, cultural background), smoking habits, alcohol intake, current use of medication, medical history and vertigo characteristics (onset, duration, frequency, triggers, associated symptoms).^6^ Additionally, the patient will be asked to fill out the shortened version of the Dizziness Handicap Inventory (DHI-S), in order to investigate impairment due to vertigo.^33^

**Table 3:** All index tests (history taking and physical examination)

**Sociodemographic characteristics**	**History taking**
*General patient characteristics*	*Vertigo characteristics*
Age	Onset
Sex	Duration
Education	Frequency
Partner status	Associated symptoms
Cultural background	Triggers
*Intoxications*	*Questionnaire*
Smoking habits	Shortened version of the Dizziness Handicap Inventory
Alcohol intake	
*Medical characteristics*	
Medication use	
Medical history	
**Physical examination**	
*Cardiovascular examination*	*Orienting neurological examination*
Pulse	Nystagmus examination
Blood pressure	Visual field examination*
Orthostatic hypotension test*	Dysarthria
Heart auscultation*	Diplopia
	Dysphagia
*Vestibular examination*	Ataxia
Dix-Hallpike manoeuvre	Walking pattern
Supine Head Roll	Sensory system
	Motor system
*ENT examination*	
Otoscopy	*Acute vestibulopathy*
	HINTS Plus exam*

*On indication.

During the physical examination, we will perform measurement of pulse and blood pressure, orthostatic hypertension test on indication (ie, vertigo provoked by standing up and combined with a tendency to fall),^6^ heart auscultation on indication (ie, vertigo provoked by exercise),^6^ nystagmus examination with Frenzel glasses, orienting neurological examination (dysarthria, diplopia, dysphagia, ataxia, walking pattern, motor system and sensory system), visual field examination on indication (ie, (partial) loss of vision),^6^ otoscopy,^6^ the Dix-Hallpike manoeuvre,^31^ the Supine Head Roll^34^ and the HINTS Plus exam on indication (ie, suspicion of vestibular neuritis, ie, subacute or acute spontaneous vertigo with nausea/vomiting and unsteadiness).^25 35^ The duration to perform all index tests will be 30–45 min, depending on the number of tests to be performed.

#### Reference standard

During this study, we will compare each index test with its respective reference standard. Researchers investigating the diagnostic accuracy of a test often encounter ‘no gold standard situations’: situations where the reference standard is not available for all patients, where the reference standard is imperfect or where there is no accepted reference standard.

Consensus or panel diagnosis, an example of a constructed reference standard, is an attractive alternative if a generally accepted reference standard does not exist and multiple sources of information have to be interpreted to reach a diagnosis. In a consensus diagnosis, a group of experts determines the presence or absence of the target condition in each patient based on multiple sources of information. These sources can include general patient characteristics, signs and symptoms from history and physical examination, and other test results.^24^

We will focus on five target conditions that account for more than 95% of vertigo diagnoses in general practice: (1) BPPV, (2) vestibular neuritis, (3) Ménière’s disease, (4) VM and (5) central causes of vertigo.^7^ As these five target conditions have a different pathophysiology and all lack a generally accepted reference standard, we will use consensus diagnosis as reference standard. Data for each patient, including history, physical examination and additional tests as recommended by international guidelines,^1 11 25 29 31 32^ will be recorded on a standardised form within Castor (see the Data collection section) and independently reviewed by a neurologist and otorhinolaryngologist. In case of disagreement, a third reviewer (neurologist or otorhinolaryngologist) will be invited to reach consensus. The reviewers will not meet, in order to avoid domination.^24^

#### Additional testing

Depending on the history taking and physical examination by the study physician during the home visit, a subset of patients may report symptoms that imply a medical indication for additional testing.

Patients with vertigo accompanied by hearing loss, tinnitus and/or aural fullness will receive pure-tone audiometry within 4 weeks after the home visit.

Patients with daily, non-episodic vertigo lasting over 14 days and substantial dizziness-related impairment (defined as a DHI-S score of ≥12)^33^ will receive state-of-the-art videonystagmography (VNG) within 4 weeks after the home visit.^36–40^ Additionally, patients indicated to undergo the HINTS Plus exam will also undergo VNG.

Prior to receiving VNG, patients will undergo a video Head Impulse Test, the Dix-Hallpike test using Frenzel glasses and the Supine Roll test using Frenzel glasses. During VNG, the function of the vestibular organ will be investigated during several test situations, that is, spontaneous nystagmus, oculomotor examination, rotational testing and caloric testing. The total testing time is about 60–90 min.

It is estimated that around 10% of patients will receive audiometry while around 30% of patients will receive VNG based on additional analyses of the general practice cohort within the DIEP (Dizziness In Elderly Patients study) study.^27^

When a patient displays a ‘red flag’, the study physician will contact the patient’s GP the same day. The study physician will advise the GP to act in accordance with the Dizziness Guideline of the Dutch College of GPs (see table 2), which implies that a patient with a red flag may receive additional testing after being referred. To get a complete testing overview, we will approach the patient’s GP to collect these additional data.

### Outcomes

For each target condition, we will quantify the level of agreement between the consensus diagnosis (see the Reference standard section) and the results obtained from individual and combinations of index tests. Subsequently, we will compute a range of diagnostic accuracy measures, including sensitivity, specificity, positive and negative predictive values, as well as positive and negative likelihood ratios, and diagnostic odds ratios (see the Statistical analysis section).

### Sample size

Given (a) the estimated distribution of causes of vertigo in general practice (ie, BPPV 42%, vestibular neuritis 41%, Ménière’s disease 10%, central causes 5%, VM unknown),^7^ (b) the estimated diagnostic accuracy of tests previously studied in secondary/tertiary care and possible feasible in general practice^22 31 41 42^ and (c) a two-sided 95% CI width of 0.20, it can be calculated that n=960 patients with vertigo should be included in the study.^43 44^ The calculated sample sizes per target condition are as follows: BPPV: n=603; vestibular neuritis: n=110; Ménière: n=189, central causes: n=960.^22 41–43^ Given the unknown prevalence of VM in general practice, it is not to possible to provide a reliable individual sample size calculation for VM.

The recruitment of 960 patients will take place during 36 months. During this period, a GP with a standard Dutch practice list size (n=2095 patients) is expected to encounter ±60 patients with vertigo.^6^ When 50% of the potential applicants in the present study will participate, we will need 960/(60*0.50)=32 standard practices to achieve the calculated sample size of 960 participants. Because some practices may enter later in the study or have the wish to participate for a shorter period of time, we will recruit 60 standard practices (or, in case of larger practice list sizes, a lower equivalent).

### Recruitment of participants

The large majority of patients will be recruited from the Academic Network of General Practices of the Amsterdam UMC (ANHA). The ANHA consists of more than 120 practices and almost 600 000 enlisted patients. The respective practices comprise a broad spectrum of practices, including both old and young practices, urban practices and practices with an over-representation of socially deprived people. When a patient with vertigo consults his/her GP, the GP will provide care as usual. At the end of the consultation, the GP will use the inclusion and exclusion criteria to assess whether the patient may be a suitable candidate to participate in the VERDI study. If the patient meets the criteria, the GP will ask if the patient is interested to receive information about the study and whether the research team has permission to contact the patient. When the patient gives permission to be contacted, the GP will hand over the patient information folder and send the patient’s contact details to the research team. The research team will call the patient and provide background information about the VERDI study, answer questions, and, when the patient has the intention to participate, schedule an appointment for a home visit after 48 hours. With a time interval of minimum 48 hours, the research group aims for an optimal balance between ‘sufficient decision time’ on the one hand (ie, enough time for the patient to decide about participation) and ‘reliable diagnosis time’ on the other hand (ie, too much time between initial symptoms and actual execution of diagnostic tests may introduce bias). After 48 hours, a trained study physician will visit the patient at home and provide background information and, if necessary, answer questions. When the patient wants to participate, the study physician will obtain a written informed consent form.

### Adverse events

Because all studied tests concern usual care and a previous cohort study among older patients (mean age 78.5 years) who received similar tests did not result in any adverse events,^27^ we consider the risk classification of this study as ‘negligible risk’. This was confirmed by the Medical Ethical Review Committee (METC). All adverse events that occur during the study, whether or not considered related, will be recorded. Serious adverse events (SAEs) will be reported to the accredited METC that approved the protocol, within 7 days of first knowledge for SAEs that result in death or are life-threatening followed by a period of maximum of 8 days to complete the initial preliminary report. All other SAEs will be reported within a period of maximum of 15 days.

### Data collection

During a home visit, the study physician will conduct data collection in which patient characteristics, history taking and physical examination are recorded in a standardised manner using the electronic case report form within the online Castor Electronic Database environment.^45^ Castor is a computer-assisted interviewing system and survey processing tool that will help to create a standardised setting and to avoid missing data. The study’s electronic case report form in Castor is programmed in such a way that it is capable of detecting alarming findings (so-called ‘red flags’) after all index tests are recorded and summarise these at the end of a home visit. In the case of missing data, these results will be imputed.

### Statistical analysis

#### Primary analysis: regression analysis and receiver operating characteristic (ROC) analysis

For each target condition, we will measure the amount of agreement between the outcome of (individual and combinations of) index tests and the consensus diagnosis. We will construct 2×2 tables to calculate sensitivity, specificity, prior probabilities, positive and negative predictive values, positive and negative likelihood ratios, and diagnostic ORs. Subsequently, we will perform multivariable logistic regression analysis with ‘presence or absence of target condition X, according to consensus diagnosis’ as dependent variable. Prior to multivariable regression analysis, we will perform univariate logistic regression analysis to investigate the associations between separate diagnostic indicators and the dependent variable. Variables will only be entered in the multivariable regression model if the univariate p value is <0.15. During multivariable analysis, we will follow the chronology in which investigations are performed in daily clinical practice.^46^ First, we will include all findings from history taking. This model will be reduced by (one by one) excluding variables with a likelihood p value of >0.10. Subsequently, we will add the results of physical examination (and possible other additional diagnostic tests) to quantify their added diagnostic value. Again, we will use a p value of >0.10 as cut-off to construct the final models. For each of the five target conditions (ie, (1) BPPV, (2) vestibular neuritis, (3) Ménière’s disease, (4) VM and (5) central causes other than VM), we will construct a final model and calculate sensitivity, specificity, predictive values, likelihood ratios, and diagnostic ORs.

After multivariable regression, in order to distinguish the discriminative ability (ie, the ability to discriminate patients with target condition X from patients without this target condition), an ROC curve will be constructed out for each target condition. An area under the curve (AUC) of 0.5 indicates no discrimination, whereas an AUC of 1.0 indicates perfect discrimination.^46^ We will test continuous variables for linear association with the outcome and we will use Spearman’s rank correlation coefficient to investigate multicollinearity.

#### Secondary analysis: reliability and validation

In order to assess the reliability of the model, we will calculate the Hosmer-Lemeshow goodness-of-fit statistic and construct calibration plots.^47^ Because diagnostic models perform better in development cohorts than in other similar populations (overfitting), we will perform a bootstrapping procedure. During this procedure, we will repeat the entire modelling process, in order to validate the final models and to adjust (shrink) the estimated performance and regression coefficients.^48^

As empirical research has shown that variability among raters influences diagnostic accuracy,^49^ we will calculate the inter-rater reliability (ie, the reliability across multiple reviewers) and intrarater reliability (ie, the reliability of a single reviewer) by calculating Cohen’s kappa. Kappa values less than 0.00 indicate poor, 0.00–0.20 slight, 0.21–0.40 fair, 0.41–0.60 moderate, 0.61–0.80 substantial and greater than 0.81 almost perfect agreement.^50^

### Data availability statement

Data from the VERDI study will be made available in the future for collaborative research questions. Such requests must be authorised by the principal investigators and the appropriate METC.

### Patient and public involvement

Patients play a crucial role during the realisation, execution and knowledge dissemination of the VERDI study. In 2020, we used the input of patients with vertigo during the writing of the research proposal. Additionally, we used the input of the Dutch patient organisation Stichting Hoormij·NVVS on diagnosing vertigo in general practice.

During the execution of the VERDI study, a patient representative of the Committee Dizziness And Balance/Hoormij·NVVS will be a permanent member of the research group in order to represent the perspective of patients with vertigo. During the interim evaluations of the VERDI study, we will ask patients from our general practice network and the patient organisation Hoormij·NVVS to provide input in order to identify possible obstacles and suggest adjustments if necessary.

Finally, we will collaborate with the patient organisation Stichting Hoormij·NVVS to successfully disseminate the results of the VERDI study to patients with vertigo.

## Discussion

### Diagnostic accuracy study

The GP’s diagnostic toolkit for vertigo is limited since recommended tests lack empirical evidence, primarily because a diagnostic accuracy study on vestibular disease has never been performed in general practice. This diagnostic accuracy study will fill this scientific gap by providing concrete accuracy measures for individual and combined signs, symptoms and diagnostic tests in adults presenting with vertigo in general practice. From a more clinical perspective, the results of our regression analyses with respect to each target condition will provide valuable insight into which clinical tests offer the largest diagnostic contribution for each specific target condition, and also which tests—based on negligible diagnostic contributions—are perhaps superfluous in vertigo diagnosis in general practice.

A possible limitation of the study is that our constructed reference standard, that is, the consensus diagnosis by a panel of experts, is dependent on results of index tests collected during the home visit by study physicians. In order to mitigate this possible limitation, all study physicians have substantial clinical expertise and will receive a 3-hour training aimed at correct execution, interpretation and documentation of the index tests. We deliberately chose not to have medical specialists carry out the index tests, since this may negatively affect the generalisability of our findings for general practice.^51^

### Following steps for the VERDI project

After this diagnostic accuracy study (phase III) has ended, the next step of VERDI will be to use the results of this study to construct a diagnostic algorithm (phase IV) in order to enable GPs to more accurately and efficiently identify underlying causes in patients with vertigo. Improvement of the current situation is crucial, because GPs on the one hand are responsible for the treatment of 80% of the patients,^7–9^ while on the other hand they are often (36–40%) not able to provide a diagnosis.^8 26^

As clinical decision rules may have limited effect on physicians—due to the perception of diminished autonomy, the conviction that clinical judgement is superior, weak incentives for using the decision rule consistently or the concern that important factors are not addressed by the decision rule^52^—it is crucial to demonstrate that a decision rule has additional value to the clinical judgement of the GP.

Using the results of a diagnostic accuracy study in order to construct a decision rule for use in clinical practice is a major scientific project which is not risk-free. Failing to convincingly demonstrate the additional value of such a clinical decision rule for GPs would result in a limited impact on clinical practice.

In phase IV of the VERDI study, we will conduct semistructured interviews with a small sample of patients and practising GPs in order to investigate barriers and facilitators for successful implementation of the decision rules and the corresponding diagnostic strategy. In the final phase of the VERDI study (phase V), we will compare the diagnostic accuracy of GP judgement with the accuracy of the constructed diagnostic algorithm.

If proven accurate, our diagnostic algorithm may lead to faster and more targeted treatment, less diagnostic imaging and referral, less prescribing of antivertigo drugs and improvement of the overall outcome for patients with vertigo in general practice. With faster and more accurate diagnoses, patients can often be treated in general practice and expensive additional testing and referral to secondary/tertiary care can be avoided.

The proposed study therefore has the potential to reduce both the enormous personal burden of vertigo and its associated economic burden (total annual economic burden in the USA is US$48.1 billion, US$13.3 billion directly attributed to vertigo/dizziness).^53^ From a wider perspective, the results of this study may contribute to further reinforcement of primary care. According to the late Barbara Starfield, this can relieve the burden on secondary/tertiary care, leading to better care at lower costs.^54^

### Ethics and dissemination

The study obtained approval from the Vrije Universiteit Medical Center Medical Ethical Review Committee (reference: 2022.0817—NL83111.029.22). We aim to widely disseminate the insights and results of this project to patients, GPs, GP trainees, physiotherapists, elderly care physicians, GP organisations and patient associations. For this purpose, we will publish the results in international peer-reviewed journals and disseminate our results by means of (national and international) conference presentations.

## Supplementary Material

Reviewer comments

Author's manuscript
