# Supplementary material for: Prospective diagnostic accuracy study of history taking and physical examination for adults with vertigo in general practice: study protocol
Source: BMJ Open. 2024 Apr 2;14(4):e085715. doi: 10.1136/bmjopen-2024-085715 (PMC10989125; doi:10.1136/bmjopen-2024-085715)
Supplement: Reviewer comments [file bmjopen-2024-085715.reviewer_comments.pdf]

PEER REVIEW HISTORY

BMJ Open publishes all reviews undertaken for accepted manuscripts. Reviewers are asked to complete a checklist review form (**http://bmjopen.bmj.com/site/about/resources/checklist.pdf**) and are provided with free text boxes to elaborate on their assessment. These free text comments are reproduced below.

ARTICLE DETAILS

**TITLE (PROVISIONAL)**	Prospective diagnostic accuracy study of history taking and physical examination for adults with vertigo in general practice: study protocol
**AUTHORS**	Ross, Andrew; Leemeyer, Anna-Marie; Bruintjes, Tjasse D.; Cals, Jochen; Bronstein, Adolfo; van Leeuwen, Roeland; Lissenberg-Witte, Birgit; van Vugt, Vincent; Rutgers, Sandra; Maarsingh, Otto

This article was not externally reviewed at BMJ Open. Protocols that have been independently assessed prior to submission to BMJ Open are usually fast-tracked to publication on the grounds that further substantial changes will not be possible. This independent assessment will usually be external, independent review for both a substantial grant award from a non-commercial or government funder and ethics approval.
